# Knowledge, Utilization Practices, and Adequacy of Iodized Salt Among Pregnant Women in Awash Sebat Administrative City, Afar Region, Ethiopia: A Bayesian Logistic Regression Analysis

**DOI:** 10.1155/jnme/1557188

**Published:** 2026-09-02

**Authors:** Temesgen Gebeyehu Wondmeneh, Asiya Hussein Ibrahim

**Affiliations:** ^1^ Department of Public Health, College of Medicine and Health Sciences, Samara University, Semera, Ethiopia, su.edu.et

**Keywords:** adequately iodized salt, Afar Region, iodized salt, knowledge, pregnant women, utilization practice

## Abstract

**Background:**

Iodine is an essential micronutrient required for thyroid hormone synthesis and fetal brain development, making pregnant women particularly vulnerable to iodine deficiency. Despite implementation of universal salt iodization in Ethiopia, adequate iodine intake remains suboptimal due to gaps in knowledge, utilization practices, and household availability of adequately iodized salt. These challenges are more pronounced in pastoral settings such as the Afar Region, where contextual factors may influence access to and use of iodized salt. Therefore, this study assessed knowledge, utilization practices, the availability of adequately iodized salt, and associated factors among pregnant women in Awash Sebat Administrative City using a Bayesian logistic regression approach.

**Methods:**

A community‐based cross‐sectional study was conducted among 411 pregnant women (response rate: 97.39%) in Awash Sebat Administrative City, Afar Region, Ethiopia, from December 1 to 30, 2024. Participants were selected using simple random sampling from health extension workers′ registration lists of pregnant women. Data were collected using a structured interviewer‐administered questionnaire and household salt iodine testing. Bayesian logistic regression was used to identify factors associated with knowledge, utilization practices, and the availability of adequately iodized salt.

**Results:**

Overall, 80.78% of pregnant women had good knowledge of iodized salt, and 62.8% demonstrated good utilization practices. Adequately iodized salt was found in nearly two‐thirds of households (62.5%). Higher odds of good knowledge of iodized salt were observed among pregnant women with college‐level or higher education (OR = 1.77; 95% CrI: 1.22–5.45), urban residence (OR = 2.48; 95% CrI: 1.57–6.32), and high wealth index (OR = 2.27; 95% CrI: 1.07–4.35). Good utilization practice was significantly associated with college‐level or higher education among pregnant women (OR = 5.08; 95% CrI: 2.70–8.03), Christian religion (OR = 2.87; 95% CrI: 1.52–4.77), and urban residence (OR = 4.58; 95% CrI: 3.43–11.52), while husband‐only decision‐making was associated with lower odds (OR = 0.21; 95% CrI: 0.11–0.53). Women with good knowledge of iodized salt had higher odds of good utilization practice (OR = 5.06; 95% CrI: 3.88–33.45). The availability of adequately iodized salt was associated with college‐level or higher education (OR = 2.13; 95% CrI: 1.10–4.20), urban residence (OR = 1.80; 95% CrI: 1.18–3.39), and a high household wealth index (OR = 2.05; 95% CrI: 1.53–4.88), whereas households with ≥ 4 members had lower odds (OR = 0.64; 95% CrI: 0.35–0.91).

**Conclusion:**

This study found that although a considerable proportion of pregnant women in Awash Sebat Administrative City had good knowledge of iodized salt, utilization practices and the availability of adequately iodized salt remained comparatively lower. Higher education, urban residence, and household wealth were consistently associated with better knowledge, utilization practices, and adequate household iodized salt. Good knowledge was positively associated with appropriate utilization practices, highlighting the importance of health education in promoting behavioral change. These findings underscore the need to strengthen community‐based nutrition education, improve access to adequately iodized salt in pastoral settings, reinforce the implementation and monitoring of universal salt iodization policies, and promote women’s participation in household decision‐making to improve iodine nutrition among pregnant women.

## 1. Introduction

Iodine is an essential micronutrient required for the synthesis of thyroid hormones, which play a critical role in human growth, neurological development, and metabolic regulation [1, 2]. Inadequate iodine intake leads to iodine deficiency disorders (IDDs), a group of conditions that affect physical growth and cognitive function and are considered the leading preventable cause of brain damage worldwide [3–5]. Globally, iodine deficiency remains a major public health problem, affecting both developed and developing countries, with an estimated 1.8 billion people at risk [6]. Pregnant women are particularly vulnerable to iodine deficiency due to increased physiological requirements needed to support fetal brain development and maternal thyroid function [7]. Insufficient iodine intake during pregnancy is associated with serious adverse outcomes, including miscarriage, stillbirth, low birth weight, impaired fetal growth, and irreversible cognitive and intellectual deficits in children [8–10]. Even mild iodine deficiency during pregnancy may negatively affect a child’s long‐term cognitive development and performance [10, 11]. Universal salt iodization (USI) is recognized by WHO as the most effective, safe, and sustainable public health strategy for the prevention and control of IDDs at the population level [12–14]. As a result, many countries, including Ethiopia, have implemented mandatory policies requiring iodization of all salt intended for human consumption as part of USI programs [12, 15, 16]. Following the implementation of USI in Ethiopia, household access to iodized salt has improved substantially. Nevertheless, evidence from the 2016 Ethiopia Demographic and Health Survey showed that although approximately 89% of households used iodized salt, only 53.9% had adequately iodized salt (≥ 15 ppm), indicating that household coverage does not necessarily translate into adequate iodine intake [17–19]. Although these estimates are derived from the most recent nationally representative Ethiopia Demographic and Health Survey available at the time of this study, they continue to provide important evidence of the gap between household access to iodized salt and the availability of adequately iodized salt. A systematic review reported a pooled prevalence of iodine deficiency of approximately 68.76% among pregnant women, highlighting a substantial burden despite high iodized salt coverage [20]. Several factors contribute to this discrepancy. Inadequate knowledge and improper utilization practices at the household level can reduce the effectiveness of iodized salt. For example, a previous study conducted in Hawassa City reported that only 52.51% and 52.33% of pregnant women had good knowledge and appropriate iodized salt utilization practices, respectively [21]. In addition, the availability of adequately iodized salt varies considerably across regions, ranging from 56.1% to 91.7% in Southern Ethiopia [22, 23] and from 29.8% to 48.1% in the Oromia Region [24, 25]. Proper utilization practices, including appropriate storage, handling, and adding salt at the end of cooking, are essential for preserving iodine content and ensuring adequate intake. In contrast, practices such as exposure to sunlight, prolonged storage, and adding salt early during cooking can significantly reduce iodine levels [24, 26, 27]. Urban residence, higher educational status, and greater household wealth were positively associated with knowledge, utilization practices, and adequate availability of iodized salt [24, 25].

There is limited context‐specific evidence on knowledge, utilization practices, and availability of adequately iodized salt among pregnant women in pastoral settings such as the Afar Region, where access to health information, market systems, and infrastructure is often constrained. These conditions may affect both the availability and proper use of adequately iodized salt. The Afar Region is Ethiopia’s principal salt‐producing area, supplying large quantities of salt harvested from the Afdera salt lake. Despite this abundant production, locally available salt is not always adequately iodized because iodization is performed after extraction through industrial processing and quality control mechanisms. Consequently, despite living near Ethiopia’s principal salt‐producing area, households may continue to consume locally produced coarse salt that is inadequately iodized or not iodized at all, thereby reducing the effectiveness of USI programs. In addition, naturally saline soil and groundwater may create misconceptions that locally available salt naturally contains sufficient iodine, thereby reducing demand for adequately iodized packaged salt. These contextual factors make the Afar Region particularly important for investigating household knowledge, utilization practices, and adequacy of iodized salt. Methodologically, community‐based surveys conducted within a limited geographic area often involve modest sample sizes, uneven distributions across categorical variables, and sparse observations in some outcome categories. Under these conditions, conventional maximum likelihood estimation may produce unstable parameter estimates and imprecise confidence intervals. Accordingly, Bayesian logistic regression was selected because it incorporates prior information through regularization, provides full probabilistic inference based on posterior distributions, and yields more stable parameter estimates with better uncertainty quantification when data are sparse or sample sizes are relatively small.

### 1.1. Research Questions

This study sought to answer the following research questions:

1. What proportion of pregnant women have good knowledge regarding iodized salt?
2. What proportion of pregnant women demonstrate good iodized salt utilization practices?
3. What proportion of households of pregnant women has adequately iodized salt (≥ 15 ppm)?
4. Which sociodemographic, socioeconomic, and behavioral factors are associated with knowledge, utilization practices, and household availability of adequately iodized salt among pregnant women?

## 2. Methods

### 2.1. Study Area and Period

The study was conducted in Awash Sebat City Administration, located in the Afar Region of northeastern Ethiopia. The region had extremely high temperatures and dry conditions. The Afar Region is predominantly pastoral with some agropastoral and is known for its extensive salt production, particularly from the Afdera area. The region is characterized by high environmental salinity, including saline soil and water sources. Awash Sebat City is situated in Zone 3 (Gabi Rasu) of the Afar Region, approximately 217 km from Addis Ababa, along the Addis Ababa–Djibouti railway line. The city comprises seven kebeles (five urban and two rural). According to local health office reports, there were an estimated 586 pregnant women residing in the city during the study period. The study was conducted from December 1 to December 30, 2024.

### 2.2. Study Design

A community‐based cross‐sectional study was conducted among pregnant women in Awash Sebat City Administration.

### 2.3. Source Population

The source population comprised all pregnant women residing in Awash Sebat City Administration during the study period who had lived in the area for at least 6 months prior to the survey.

### 2.4. Study Population

The study population consisted of pregnant women living in households selected from the sampled kebeles of Awash Sebat City Administration, who were available during the data collection period and fulfilled the eligibility criteria.

### 2.5. Inclusion and Exclusion Criteria

Pregnant women were included in the study if they had lived in Awash Sebat City Administration for 6 months or more, were identified as pregnant at the time of data collection by health extension workers, and provided informed consent to participate in the study. Pregnant women who were seriously ill or unable to communicate during the data collection period, as well as those with medical conditions requiring salt restriction (e.g., chronic hypertension), were excluded from the study.

### 2.6. Sample Size Determination

The sample size was determined using the single population proportion formula with OpenEpi Version 3.01 software, assuming a 95% confidence level, a 5% margin of error, and a proportion (p) of 48.1% for adequately iodized salt availability reported from a previous Ethiopian study conducted in North Shoa Zone, Oromia Region (41). This estimate was selected because, at the time of study planning, no comparable published study was available from the Afar Region. Although a conservative estimate of 50% could also have been used, the selected proportion represented the best available evidence from a geographically comparable Ethiopian setting and resulted in a similar required sample size. After adding a 10% nonresponse rate, the final sample size was 422.

### 2.7. Sampling Procedure

**FIGURE 1:**
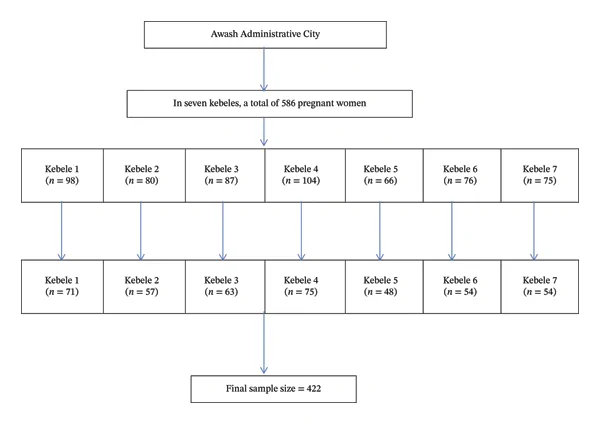
Sampling procedure of study participants.

The sampling procedure was conducted through a series of systematic steps. Initially, a complete sampling frame was established by obtaining a list of all registered pregnant women (*N* = 586) from the health extension workers’ registration books across the seven kebeles of Awash 7 City. The lists were thoroughly reviewed to remove duplicate and incomplete records, ensuring accuracy and completeness. Following this, the final sample size (*n* = 422) was proportionally allocated to each kebele based on the number of registered pregnant women to ensure representativeness. The allocated sample sizes for each kebele were determined accordingly. Subsequently, all eligible pregnant women in each kebele were assigned unique identification numbers. A simple random sampling technique was then applied using a computer‐generated random number method to select the required number of participants from each kebele. Finally, the selected participants were traced to their respective households with the assistance of health extension workers and kebele administrators. Data collectors visited each household to conduct face‐to‐face interviews and perform household salt testing. The sampling frame relied on health extension worker registration records; therefore, pregnant women who were not registered or had recently migrated into the study area may not have been included in the sampling frame. This potential source of selection bias was considered when interpreting the findings and is acknowledged as a study limitation (Figure 1).

### 2.8. Data Collection Tools and Procedures

Data were collected using a pretested, structured, interviewer‐administered questionnaire adapted from relevant literature and previous studies [21–27]. The questionnaire was administered electronically using KoboToolbox and consisted of four sections: sociodemographic characteristics, knowledge of iodized salt, utilization practices of iodized salt, and adequacy of iodized salt. Data were collected by seven trained female Bachelor of Science degree holders in Clinical Laboratory Science who were fluent in the Amharic and Afar languages. Two supervisors holding Master of Science degrees in Clinical Chemistry and experienced in supervising similar studies oversaw the data collection process. Before the commencement of data collection, participants were informed about the purpose and significance of the study, and informed consent was obtained. Data collectors conducted face‐to‐face interviews with eligible participants. If a selected participant was not available during the initial visit, revisits were conducted. When an eligible respondent could not be found after repeated visits, the household was considered nonresponsive.

### 2.9. Variables and Measurements

#### 2.9.1. Sociodemographic Variables

The sociodemographic variables included age of the mother, residence, religion, ethnicity, marital status, occupation of the mother, occupation of the husband, educational status of the mother, educational status of the husband, household wealth index category, household size, and decision‐making power on household income utilization.

#### 2.9.2. Wealth Index Measurement

Data on the household wealth index were collected using standard Demographic and Health Survey (DHS) questionnaires. A total of 16 asset‐related variables, including ownership of durable assets, housing characteristics, and access to basic services, were recoded into binary or categorical variables. Household characteristics such as sources of drinking, cooking, and washing water, type of toilet facility, and housing materials (roof and floor) were classified as improved or unimproved, while cooking fuel type was categorized as clean or solid fuel based on DHS classification. Principal component analysis (PCA) was used to construct the household wealth index. The first principal component was used to generate wealth scores, which were then categorized into tertiles as low, medium, and high wealth groups.

### 2.10. Knowledge of Iodized Salt

Knowledge was assessed using seven structured questions covering awareness of iodized salt, sources of information, benefits of iodine during pregnancy, consequences of IDDs, and appropriate iodized salt use [21–27].

### 2.11. Utilization Practices of Iodized Salt

Utilization practices were assessed using seven recommended household practices related to purchase, storage, handling, and cooking of iodized salt, including timing of salt addition, packaging status, storage conditions, exposure to sunlight, storage location, and duration of storage [21–27].

### 2.12. Adequacy of Iodized Salt

The adequacy of iodized salt was assessed at the household level using a rapid test kit (RTK).

### 2.13. Salt Iodine Testing and Measurement Procedure

A tablespoon of salt was collected from each selected household, and iodine content was determined using a RTK. The RTK is a field‐based tool used to assess salt fortified with potassium iodide or potassium iodate, with reported sensitivity and specificity of 89.8% and 65.8%, respectively. The kit consists of two white ampoules (each containing 10 mL of test solution), one red ampoule (10 mL recheck solution), a plastic testing cup, a color comparison chart indicating 0 ppm, < 15 ppm, and ≥ 15 ppm iodine levels, and an instruction manual. For testing, the salt sample was placed in the cup and leveled. Two drops of test solution from a white ampoule were added by piercing and gently squeezing the ampoule. The iodine level was determined within 1 min by comparing the color change with the standard chart. If no color appeared after 1 min, five drops of the recheck solution from the red ampoule were added to a fresh salt sample, followed by two drops of the test solution, and the result was again compared with the standard chart. Although the RTK has acceptable field sensitivity (89.8%), its specificity (65.8%) may result in some inadequately iodized salt samples being misclassified as adequately iodized. Because laboratory‐based iodometric titration was not feasible under field conditions, confirmatory laboratory testing could not be performed. Therefore, the findings related to salt adequacy should be interpreted with this limitation in mind.

### 2.14. Outcome Variable Measurements (Operational Definitions)

#### 2.14.1. Knowledge of Iodized Salt

Participants scoring 50% or more of the knowledge questions were classified as having good knowledge, whereas those scoring below 50% were classified as having poor knowledge. The 50% cutoff was adopted from previous Ethiopian studies assessing iodized salt knowledge and utilization practices [21–27], where the same operational definition was used. Although no universally accepted threshold exists for classifying knowledge or utilization practices, use of this cutoff facilitates comparability with previous Ethiopian studies.

#### 2.14.2. Utilization Practices of Iodized Salt

Households correctly performing at least 50% of the recommended iodized salt utilization practices were classified as having good utilization practices. This cutoff was similarly adopted from previous Ethiopian studies evaluating iodized salt utilization practices [21–27].

#### 2.14.3. Adequately Iodized Salt

Salt samples with iodine content ≥ 15 parts per million (ppm) were classified as adequately iodized, while those with < 15 ppm were considered inadequately iodized.

### 2.15. Data Quality Control

Data were collected using KoboToolbox‐based electronic data collection tools. One‐day training was provided to data collectors and supervisors on the study objectives, interview techniques, and proper completion of the questionnaire. The principal investigator and supervisors closely monitored the data collection process on a daily basis to ensure completeness, consistency, and accuracy of the collected data. Completed questionnaires were reviewed daily, and any incomplete or inconsistent entries were corrected through immediate follow‐up or revisits. To ensure data validity and linguistic consistency, the questionnaire was initially prepared in English, translated into Afar and Amharic languages, and then back‐translated into English by language experts and the principal investigator. A pretest was conducted on 5% of the sample size (21 participants) in a nonselected kebele of Awash Arba to assess clarity, validity, and consistency of the data collection tool. Necessary modifications were made based on the pretest findings. Data were checked for completeness, accuracy, and consistency before analysis. Proper coding and categorization were maintained throughout the data handling process. For salt iodine measurement, a rapid iodine test kit (RTK) was used within its valid expiry period to ensure accurate determination of iodine content.

### 2.16. Data Processing and Analysis

Data were exported from Kobo Toolbox and analyzed using Stata Version 17. Descriptive statistics were used to summarize participants’ characteristics.

Bayesian logistic regression was applied separately for the three binary outcomes. Separate models were fitted because each outcome represented a distinct public health construct with different determinants. Since these analyses addressed independent research questions rather than repeated testing of the same hypothesis, adjustment for multiple comparisons was not performed, consistent with recommendations for explanatory epidemiological analyses [28, 29]. Weakly informative normal priors [*N* (0, 10^2^)] were assigned to all regression coefficients to provide mild regularization while allowing the observed data to dominate posterior estimation [30]. To evaluate the robustness of the Bayesian models, sensitivity analyses were conducted using alternative weakly informative priors [*N* (0, 5^2^) and *N* (0, 20^2^)]. Posterior estimates remained materially unchanged across prior specifications, indicating that the findings were robust to reasonable prior assumptions [31, 32]. Model adequacy was assessed using posterior predictive checks, effective sample sizes (ESS), Monte Carlo standard errors (MCSEs), and the widely applicable information criterion (WAIC), which was used to compare competing model specifications [33–35].

#### 2.16.1. Model Specification

For each outcome, the response variable Yi followed a Bernoulli distribution with probability *p*_*i*_, and a logit link function was used to model the relationship between the outcome and explanatory variables.

#### 2.16.2. Convergence Diagnostics and Model Adequacy

Convergence and adequacy of the Bayesian logistic regression models were assessed for all three outcome variables (knowledge of iodized salt, utilization practice, and availability of adequately iodized salt) using multiple complementary approaches. Visual inspection of trace plots was performed to evaluate the mixing and stability of the Markov chain Monte Carlo (MCMC) chains, with stable chains showing random fluctuations around a constant mean and no apparent trends or drifts. The Gelman–Rubin convergence diagnostic (*Ȓ*) was used to quantitatively assess convergence, with *Ȓ* < 1.1 [36] considered indicative of satisfactory convergence. ESS and MCSE [37] were also examined to evaluate the precision and stability of posterior estimates. Autocorrelation plots were inspected to assess the independence of successive MCMC samples, with decreasing autocorrelation across increasing lags indicating acceptable independence [38]. Model adequacy was assessed using posterior predictive checks, including visual inspection of posterior predictive density plots and observed‐versus‐simulated summaries. Good agreement between observed and simulated data indicated adequate model fit and calibration.

Candidate variables were selected based on prior epidemiological evidence, biological plausibility, and causal relevance rather than statistical significance alone. Multicollinearity among independent variables was assessed using the variance inflation factor (VIF).

## 3. Results

### 3.1. Sociodemographic Characteristics of the Study Participants

A total of 411 pregnant women participated in this study, yielding a response rate of 97.39%. The mean age of the participants was 27.82 years (SD ± 5.9). Among them, 30.66% were in the age group of 23–27 years. The vast majority (96.84%) of the participants were urban residents. Most respondents (74.94%) were followers of the Muslim religion, and 60.84% belonged to the Afar ethnic group. About 89.54% of the women were married or living with their husbands. Nearly half of the participants (48.05%) were housewives. Only 4.14% of the pregnant women were unable to read and write, and 35.52% of the participants were from households with a low wealth index. The majority of households (87.8%) had fewer than four members. About 22.01% of husbands were agro‐pastoralists, and 54.89% had primary education. About 60.3% of household financial decisions were made by husbands. All pregnant women had antenatal care follow‐up (Table 1).

**TABLE 1:** Sociodemographic characteristics of Awash Sabat City Administration, Afar, Ethiopia, 2024.

Variables	Category	Frequency	Percentage (%)
Age of mother	18–22	90	21.9
	23–27	126	30.66
	28–32	98	23.84
	33–37	56	13.63
	38–42	41	9.98

Residence	Urban	398	96.84
	Rural	13	3.16

Religion of mother	Muslim	308	74.94
	Orthodox	98	23.84
	Protestant	5	1.22

Ethnicity	Afar	248	60.84
	Amhara	83	20.19
	Oromo	38	9.25
	Tigre	42	10.22

Marital status	Married/living together	368	89.54
	Divorced	43	10.46

Mother’s occupation	Government employee	144	35.12
	Housewife	197	48.05
	Private employee	69	16.83

Husband’s occupation	Agro‐pastoralist	81	22.01
	Government employee	244	66.30
	Private employee	43	11.68

Mother’s education	Unable to read and write	17	4.14
	Primary education (1–8)	225	54.74
	Secondary education (9–12)	109	26.52
	College and above	60	14.6

Husband’s education	Unable to read and write	6	1.63
	Primary education (1–8)	202	54.89
	Secondary education (9–12)	106	28.8
	College and above	54	14.67

Wealth index category	Low	146	35.52
	Medium	135	32.85
	High	130	31.63

Household size	< 4	360	87.8
	≥ 4	50	12.2

Household money decision‐making	Both jointly	122	29.76
	Husbands	248	60.3
	Wives	40	9.76

### 3.2. Knowledge of Pregnant Women on Iodized Salt

Among the study participants, less than half (46.47%) had heard about iodized salt, with health workers being the most common source of information (42.41%). Approximately seven in 10 participants knew the importance of increased iodine intake during pregnancy (69.10%) and the benefits of using iodized salt during cooking (71.29%). Among women who recognized the health consequences of iodine deficiency, stunted growth (52.0%) and malformation (22.15%) were the most frequently identified conditions, whereas relatively few identified goiter (11.39%), mental retardation (8.31%), or miscarriage (6.15%). Overall, 80.78% of pregnant women demonstrated good knowledge of iodized salt. Because the knowledge score was derived from multiple items assessing iodine nutrition, the benefits of iodized salt, IDDs, and utilization practices rather than solely from awareness of the term “iodized salt,” the proportion classified as having good knowledge exceeded the proportion who reported having previously heard of iodized salt (Table 2).

**TABLE 2:** Knowledge characteristics of pregnant women about iodine and iodized salt utilization at Awash Sabat City Administration, Afar, Ethiopia, 2024.

Variables	Category	Frequency	Percentage
Heard about iodized salt	Yes	191	46.47
	No	220	53.53

Source of information	Television	60	31.41
	Health workers	81	42.41
	Radio	11	5.76
	Friends	21	11
	Printed material	18	9.42

Know the importance of an increased intake of iodine during pregnancy	Yes	284	69.10
	No	127	30.90

Know the benefits of cooking food with iodized salt	Yes	293	71.29
	No	118	28.71

Which benefit of iodine do you know?	Prevents goiter	64	21.84
	Promotes good fetal growth	77	26.28
	Promotes health	100	34.13
	Prevents miscarriage	52	17.75

Know the problems caused by loss of iodine from the diet we eat	Yes	325	79.08
	No	86	20.92

What problems of iodine deficiency did you know?	Goiter	37	11.39
	Mental retardation	27	8.31
	Miscarriage	20	6.15
	Stunted growth	169	52
	Malformation	72	22.15

Overall knowledge score	Poor	79	19.22
	Good	332	80.78

### 3.3. Practice of Utilization of Iodized Salt

Most participants reported appropriate iodized salt handling practices, including adding salt at the end of cooking (95.38%), storing salt in covered containers (92.46%), and storing it in a cool, dry place (75.70%). Overall, 62.8% of pregnant women demonstrated good iodized salt utilization practices, which was notably lower than the proportion with good knowledge of iodized salt (80.78%), indicating a substantial knowledge–practice gap (Table 3).

**TABLE 3:** Practice of the utilization of iodized salt by pregnant women at Awash Sabat City Administration, Afar, Ethiopia, 2024.

Variable	Category	Frequency	Percentage
When you add salt to the food while cooking?	At the end of cooking	392	95.38
	At the middle of cooking	19	4.62

Where did you usually purchase salt?	Open market or small shops	379	92.21
	Super market	32	7.79

Was the salt you purchased in its original package?	Yes	390	94.89
	No	21	5.11

Is your salt stored at home covered?	Yes	380	92.46
	No	31	7.54

Was the salt exposed to sunlight?	Yes	118	28.7
	No	293	71.3

Where was salt usually stored?	Dry and cool place	311	75.7
	Humid/near fire area	100	24.3

For how long did you store salt once you bought?	≤ 2 months	289	70.32
	> 2 months	122	29.68

Overall utilization practice of iodized salt	Poor	153	37.2
	Good	258	62.8

### 3.4. Adequate Iodized Salt Among Pregnant Women’s Household at Awash Sabat City Administration, Afar, Ethiopia, 2024

**FIGURE 2:**
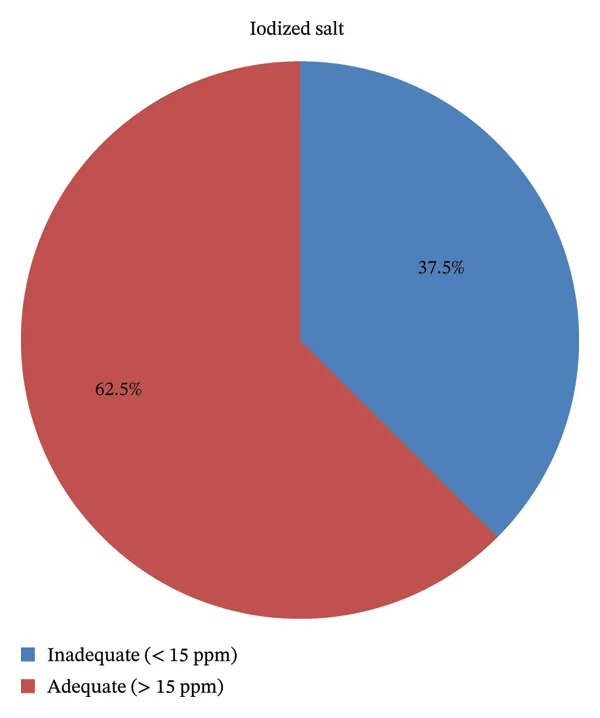
Proportion of adequately iodized salt among households of pregnant women in Awash Sebat City Administration, Afar Region, Ethiopia, 2024.

Among households with pregnant women, adequately iodized salt was identified in nearly two‐thirds of households (62.5%; 95% CI: 57.7–67.1) based on RTK measurements. Because the RTK has limited specificity, this estimate should be interpreted with caution, as some inadequately iodized salt samples may have been misclassified as adequately iodized (Figure 2).

### 3.5. Model Convergence and Adequacy

All Bayesian logistic regression models fitted for knowledge of iodized salt, utilization practice, and adequately iodized salt demonstrated satisfactory convergence. Visual inspection of trace plots from the four MCMC chains showed good mixing and stable convergence, with no apparent trends or drifts, indicating adequate exploration of the posterior distribution. The Gelman–Rubin diagnostic statistics (*Ȓ*) were close to 1 across all models, with mean values of 1.01 (range: 1.00–1.06) for knowledge, 1.03 (range: 1.00–1.09) for utilization practice, and 1.02 (range: 1.00–1.07) for availability of adequately iodized salt. ESSs ranged from 1050 to 3500 for knowledge, 1000 to 3100 for utilization practice, and 1200 to 3800 for availability, indicating adequate precision of the posterior estimates. Autocorrelation decreased with increasing lag in all models, suggesting acceptable independence of posterior samples. MCSEs were small relative to posterior standard deviations, indicating stable estimates. Model adequacy, assessed using posterior predictive checks, showed good agreement between observed and predicted values, with Bayesian *p* values of 0.49 for knowledge, 0.47 for utilization practice, and 0.50 for availability of adequately iodized salt, indicating adequate model fit. MCMC diagnostic plots (trace, histogram, autocorrelation, and posterior density plots) for knowledge of iodized salt, utilization practices, and adequately iodized salt are provided in the Supporting Information as S Figures 1–6, S Figures 7–13, and S Figures 14–21, respectively.

### 3.6. VIFs

Multicollinearity was assessed using the VIF and Pearson correlation analysis. The VIF results indicated moderate multicollinearity between maternal education (VIF = 7.09) and husband education (VIF = 6.48), while all other variables had VIF values below 5, with a mean VIF of 2.63. Pearson correlation analysis further showed a strong correlation between maternal and husband education (*r* = 0.8064, *p* < 0.001), confirming the presence of multicollinearity. Therefore, husband education was excluded from the multivariable analysis, and maternal education was retained based on its theoretical relevance.

### 3.7. Factors Associated With Knowledge of Iodized Salt Among Pregnant Women in Awash Sebat City, Afar Region, Ethiopia

Pregnant women with college‐level or higher education had significantly higher odds of having good knowledge of iodized salt compared to those with no formal education (OR = 1.77; 95% CrI: 1.22–5.45). However, the relatively wide credible interval suggests greater uncertainty regarding the precise magnitude of the estimated effect. Urban residents also had higher odds of good knowledge compared to rural residents (OR = 2.48; 95% CrI: 1.57–6.32). Similarly, pregnant women from households with a high wealth index had higher odds of good knowledge of iodized salt compared to those from low‐wealth households (OR = 2.27; 95% CrI: 1.07–4.35) (Table 4).

**TABLE 4:** Factors associated with knowledge of iodized salt among pregnant women.

Variable	Category	Odds ratio (posterior mean)	Std. dev.	MCSE	Median	95% credible interval
Age (years)	18–22	Reference	—	—	—	—
	23–27	1.54	0.56	0.007	1.45	0.73–2.89
	28–32	1.39	0.59	0.007	1.28	0.58–2.83
	33–37	0.99	0.54	0.007	0.87	0.35–2.35
	38–42	1.36	0.75	0.01	1.20	0.44–3.192

Maternal education	No education	Reference	—	—	—	—
	Primary	1.13	0.9	0.013	0.88	0.32–3.51
	Secondary	0.92	0.64	0.01	0.77	0.18–2.57
	College and above	1.77	1.41	0.02	1.56	1.22–5.45

Ethnicity	Afar	Reference	—	—	—	—
	Amhara	1.39	0.78	0.011	1.21	0.44–3.36
	Oromo	1.17	0.76	0.011	0.99	0.28–3.08
	Tigray	2.24	1.6	0.025	1.79	0.54–6.48

Religion	Muslim	Reference	—	—	—	—
	Christian	2.22	1.110	0.014	1.100	0.8–5.02

Residence	Rural	Reference	—	—	—	—
	Urban	2.48	1.523	0.022	2.178	1.57–6.32

Wealth index	Low	Reference	—	—	—	—
	Medium	2.02	0.728	0.009	1.9	0.98–3.78
	High	2.27	0.850	0.011	2.12	1.07–4.35

Mother’s occupation	Housewife	Reference	—	—	—	—
	Government employee	1.05	0.408	0.005	0.98	0.46–2.03
	Private employee	1.82	1.09	0.016	1.55	0.58–4.66

Husband’s occupation	Agro‐pastoralist	Reference	—	—	—	—
	Government employee	1.17	0.441	0.006	1.083	0.58–2.27
	Private employee	1.86	1.18	0.017	1.55	0.58–4.93

Household size	< 4	Reference	—	—	—	—
	≥ 4	1.75	0.919	0.012	1.547	0.65–4.08

Financial decision‐making	Joint	Reference	—	—	—	—
	Husband only	1.23	0.59	0.008	1.099	0.47–2.73
	Wife only	1.34	0.62	0.01	1.11	0.58–2.83

### 3.8. Factors Associated With Utilization Practices of Iodized Salt

Pregnant women with college‐level or higher education had significantly higher odds of good iodized salt utilization practices compared to those with no formal education (OR = 5.08; 95% CrI: 2.70–8.03). Christian pregnant women had higher odds of good utilization practices compared to Muslim pregnant women (OR = 2.87; 95% CrI: 1.52–4.77). Similarly, urban pregnant women had significantly higher odds of good utilization practices compared to rural pregnant women (OR = 4.58; 95% CrI: 3.43–11.52). Conversely, pregnant women in households where husbands alone made financial decisions had significantly lower odds of good iodized salt utilization practices compared to those with joint decision‐making (OR = 0.21; 95% CrI: 0.11–0.53). Pregnant women with good knowledge of iodized salt had substantially higher odds of good utilization practices compared to those with poor knowledge (OR = 5.06; 95% CrI: 3.88–33.45). The relatively wide credible interval for this association suggests some uncertainty in the estimated effect size, likely reflecting the relatively small number of participants classified as having poor knowledge (Table 5).

**TABLE 5:** Factors associated with utilization practices of iodized salt.

Variable	Category	Odds ratio (posterior mean)	Std. dev.	MCSE	Median	95% credible interval
Age	18–22	Reference	—	—	—	—
	23–27	2.03	2.03	0.017	1.97	0.62–6.51
	28–32	3.72	4.32	0.037	3.10	0.90–13.58
	33–37	1.63	0.62	0.007	1.53	0.75–3.11
	38–42	6.39	1.401	0.144	5.90	0.58–14.32

Maternal education	No education	Reference	—	—	—	—
	Primary	0.84	0.76	0.010	0.82	0.01–2.45
	Secondary	0.97	0.99	0.008	0.81	0.03–3.28
	College+	5.08	1.148	0.126	4.90	2.70–8.03

Ethnicity	Afar	Reference	—	—	—	—
	Amhara	1.05	0.56	0.007	0.98	0.38–2.38
	Oromo	0.88	0.49	0.005	0.86	0.27–2.05
	Tigray	1.06	0.89	0.010	1.02	0.17–3.11

Religion	Muslim	Reference	—	—	—	—
	Christian	2.87	1.41	0.173	2.70	1.52–4.77

Residence	Rural	Reference	—	—	—	—
	Urban	4.58	2.10	0.336	4.20	3.43–11.52

Wealth index	Low	Reference	—	—	—	—
	Medium	0.65	0.48	0.006	0.70	0.03–1.40
	High	0.45	0.34	0.004	0.51	0.01–1.09

Mother’s occupation	Housewife	Reference	—	—	—	—
	Government	0.71	0.50	0.006	0.77	0.04–1.55
	Private	1.09	0.86	0.009	1.17	0.08–2.56

Husband’s occupation	Agro‐pastoral	Reference	—	—	—	—
	Government	0.81	0.62	0.007	0.83	0.05–2.00
	Private	1.66	2.05	0.022	1.60	0.33–6.96

Household size	< 4	Reference	—	—	—	—
	≥ 4	0.84	0.32	0.003	0.79	0.38–1.60

Financial decision‐making	Joint	Reference	—	—	—	—
	Husband only	0.21	1.17	0.140	0.25	0.11–0.53
	Wife only	1.50	0.40	0.23	1.45	0.26–2.10

Knowledge of iodized salt	Poor	Reference	—	—	—	—
	Good	5.06	6.23	0.062	4.72	3.88–33.45

### 3.9. Associated Factors of Adequately Iodized Salt Among Households of Pregnant Women

Pregnant women with college‐level or higher education had higher odds of having adequately iodized salt in their households compared to those with no formal education (OR = 2.13; 95% CrI: 1.10–4.20). Similarly, urban residents were more likely to have adequately iodized salt at the household level than rural residents (OR = 1.80; 95% CrI: 1.18–3.39). Pregnant women from households with a high wealth index had significantly higher odds of having adequately iodized salt compared to those in the low‐wealth category (OR = 2.05; 95% CrI: 1.53–4.88). In contrast, households with four or more members were less likely to have adequately iodized salt compared to smaller households (OR = 0.64; 95% CrI: 0.35–0.91) (Table 6).

**TABLE 6:** Associated factors of adequately iodized salt among households of pregnant women.

Variable	Category	Odds ratio (posterior mean)	Std. dev.	MCSE	Median	95% CrI
Age (years)	18–22	Reference	—	—	—	—
	23–27	0.94	0.26	0.0023	0.89	0.57–1.54
	28–32	1.12	0.38	0.0035	1.04	0.63–2.00
	33–37	1.08	0.46	0.0052	1.01	0.52–2.08
	38–42	0.92	0.31	0.0026	0.89	0.48–1.72

Education	No education	Reference	—	—	—	—
	Primary	1.12	0.36	0.0043	1.07	0.58–1.98
	Secondary	0.91	0.31	0.003	0.85	0.5–1.62
	College+	2.13	0.84	0.0053	1.94	1.1–4.2

Ethnicity	Afar	Reference	—	—	—	—
	Amhara	0.99	0.29	0.0036	0.95	0.56–1.66
	Oromo	0.98	0.39	0.0037	0.92	0.43–1.86
	Tigray	1.03	0.38	0.0028	0.96	0.53–2.00

Religion	Muslim	Reference	—	—	—	—
	Christian	1.50	0.47	0.0037	1.44	0.82–2.54

Residence	Rural					
	Urban	1.80	0.75	0.0081	1.69	1.18–3.39

Wealth	Low	Reference	—	—	—	—
	Medium	1.0	0.25	0.002	0.97	0.61–1.56
	High	2.05	0.15	0.0011	1.99	1.53–4.88

Mother’s occupation	Housewives	Reference	—	—	—	—
	Govt	1.28	0.4	0.0032	1.26	0.65–2.04
	Private	1.44	0.42	0.0036	1.38	0.77–2.41

Husband’s occupation	Agro‐pastoral	Reference	—	—	—	—
	Govt	1.04	0.28	0.0022	1.01	0.61–1.67
	Private	0.80	0.25	0.0021	0.79	0.38–1.33

Household size	< 4	Reference	—	—	—	—
	≥ 4	0.64	0.19	0.002	0.62	0.35–0.91

Decision	Joint	Reference	—	—	—	—
	Husbands	1.31	0.44	0.0029	1.20	0.77–2.32
	Wives	1.51	0.47	0.0038	1.46	0.82–2.44

Knowledge	Poor	Reference	—	—	—	—
	Good	1.47	0.37	0.0033	1.43	0.87–2.29

## 4. Discussion

The present study assessed knowledge, utilization practices, and the availability of adequately iodized salt among pregnant women in Awash Sebat Administrative City, Afar Region, Ethiopia, using a Bayesian logistic regression approach. The findings highlight a substantial gap between knowledge and utilization practices, with good knowledge being considerably more common than appropriate utilization practices, as well as a persistent gap between utilization practices and the household availability of adequately iodized salt. These findings suggest that improving knowledge alone is insufficient to ensure appropriate household utilization of iodized salt or adequate iodine intake.

In this study, 80.78% of pregnant women demonstrated good knowledge of iodized salt. This is considerably higher than the 52.51% reported in Hawassa City [21]. The difference may be explained by strengthened iodized salt promotion programs over time, improved local implementation in Awash Sebat, and increased exposure to health information among pregnant women in the study area. Given that this study was conducted in a single administrative city, the findings should be interpreted within the local context rather than in relation to the global burden of iodine deficiency. Nevertheless, the coexistence of a relatively high level of knowledge (80.78%) with lower levels of good utilization practices (62.8%) and household availability of adequately iodized salt (62.5%) suggests that knowledge alone does not necessarily translate into appropriate household practices.

An apparent discrepancy was observed between the proportion of women who reported having heard of iodized salt (46.47%) and those classified as having good knowledge (80.78%). This discrepancy is likely attributable to the study’s operational definition of knowledge. Knowledge was assessed using multiple questions addressing the importance of iodine during pregnancy, the benefits of iodized salt, IDDs, and appropriate utilization practices, rather than a single question on whether participants had heard of iodized salt. Consequently, some women who did not explicitly recognize the term “iodized salt” may nevertheless have demonstrated good knowledge of iodine nutrition through their responses to other knowledge items. Alternatively, the discrepancy may reflect differences in how health information is communicated across sources. For example, health workers may emphasize the health benefits of iodine during pregnancy without consistently using the specific term “iodized salt,” whereas information obtained through television, radio, friends, or printed materials may vary in both accuracy and completeness. However, because the content and quality of information from each source were not assessed in the present study, this explanation remains speculative and warrants further investigation. Another noteworthy finding was that participants most frequently identified stunted growth and congenital malformation as consequences of iodine deficiency, whereas relatively few recognized goiter, despite it being one of the classical IDDs. This pattern may reflect differences in the content or emphasis of health education messages delivered through various information sources. Because this study did not evaluate the accuracy or completeness of information provided by television, radio, printed materials, friends, or health workers, no conclusions can be drawn regarding the quality of these information sources. Future studies should assess both the content and effectiveness of different communication channels in improving comprehensive knowledge of IDDs.

The prevalence of good utilization practices (62.8%) was also higher than that reported in Hawassa City (52.33%) [21], suggesting improvement in household salt handling practices. Nevertheless, this level remains below optimal considering the importance of appropriate storage and adding salt after cooking to minimize iodine loss [24, 26, 27]. The marked difference between knowledge (80.78%) and utilization practices (62.8%) indicates that knowledge alone is insufficient to ensure appropriate household behavior. Although women may understand the health benefits of iodized salt, the translation of knowledge into practice may be hindered by multiple barriers, including limited access to adequately iodized packaged salt, preference for locally available coarse salt, economic constraints, cultural cooking practices, inadequate household purchasing power, and limited participation of women in household purchasing and food preparation decisions. In addition, market availability and the affordability of adequately iodized packaged salt may constrain the adoption of recommended practices despite adequate awareness. These findings suggest that health education should be complemented by interventions addressing behavioral, social, and structural barriers.

The proportion of households with adequately iodized salt (62.5%) in this study was comparable to the range reported by previous Ethiopian studies conducted in different settings [22–25]. Because some of the national estimates cited in earlier studies [17–19] are more than a decade old, direct comparisons should be interpreted cautiously, as substantial improvements in USI coverage may have occurred over time. The observed differences across studies may reflect variations in study period, geographical setting, market access, enforcement of iodization regulations, and salt distribution systems. Therefore, temporal differences, in addition to contextual factors, should be considered when interpreting these comparisons. Furthermore, this estimate should be interpreted cautiously because the RTK used has relatively low specificity, which may have resulted in some misclassification of inadequately iodized salt as adequately iodized. Although confirmatory laboratory testing was not feasible because of resource limitations, the RTK remains the standard field method recommended for population‐based household surveys. In the Afar Region, where salt is naturally abundant and locally produced coarse salt is commonly available, the use of non‐iodized or inadequately iodized coarse salt may partly explain the proportion of households without adequately iodized salt. Although the present study did not distinguish between locally produced coarse salt and commercially packaged iodized salt because the questionnaire used closed‐ended response options, this remains an important contextual factor that should be investigated in future studies.

Overall, the findings of this study are consistent with the broader literature emphasizing the importance of universal salt iodization as a key strategy for preventing IDDs [12–14]. However, the results also demonstrate that achieving adequate iodine intake requires not only the availability of iodized salt but also proper knowledge, utilization practices, and supportive socioeconomic conditions. The discrepancies observed between this study and previous findings may be attributed to differences in study settings, population characteristics, and methodological approaches, including the use of Bayesian analysis, which provides more robust estimates by incorporating prior information and accounting for uncertainty. However, some Bayesian estimates, particularly those for college‐level education and the association between knowledge and utilization practices, were accompanied by relatively wide credible intervals, indicating considerable uncertainty around the magnitude of these associations. This imprecision likely reflects the relatively small number of participants in some exposure categories, especially women with no formal education and those classified as having poor knowledge. Therefore, while the direction of these associations is consistent with previous evidence, caution is warranted when interpreting the precise magnitude of the estimated effects.

Pregnant women with college‐level or higher education had higher odds of good knowledge, good utilization practices, and adequate iodized salt availability. This finding is consistent with previous studies that identify education as a key determinant of knowledge and health‐related behavior [24, 25]. Educated women are more likely to access, understand, and apply relevant information, including the importance of iodine during pregnancy and proper salt handling practices. This is particularly important given the well‐documented adverse effects of iodine deficiency during pregnancy, such as miscarriage, stillbirth, and impaired cognitive development in children [8–10].

Urban residence was positively associated with good knowledge, appropriate utilization practices, and the household availability of adequately iodized salt, consistent with previous studies [24, 25]. However, because 96.84% of participants were urban residents, these findings should not be generalized to predominantly rural or pastoral populations in the Afar Region, where barriers related to market access, transportation, and access to health information may differ. In addition, the small proportion of rural participants (3.16%) limited the precision of the residence‐related estimates. Therefore, future studies should include larger and more representative rural and pastoral populations to provide more reliable estimates and improve the generalizability of the findings.

The household wealth index was also significantly associated with knowledge and adequate iodized salt availability, consistent with earlier findings [24, 25]. Wealthier households are more likely to afford packaged iodized salt and maintain appropriate storage conditions, which helps preserve iodine content. In contrast, households with lower socioeconomic status may rely on cheaper, non‐iodized, or inadequately iodized salt, contributing to persistent iodine deficiency.

Christian households had higher odds of good utilization practices compared to Muslim households. Although this association was statistically significant, it should be interpreted cautiously because religion itself is unlikely to be the direct determinant of iodized salt utilization. Rather, this finding may reflect unmeasured contextual differences such as differences in educational attainment, household socioeconomic status, food preparation traditions, gender roles in household food management, or differential exposure to antenatal nutrition counseling and health extension services rather than religion itself. Because these factors were not directly measured in the present study, no causal interpretation should be made. Additional qualitative or mixed‐methods research is warranted to better understand this association.

Additionally, households where decisions were made solely by husbands had significantly lower odds of good utilization practices. This finding underscores the importance of women’s autonomy in household health‐related decisions, as joint decision‐making may facilitate better adoption of recommended practices. This finding has important programmatic implications. Nutrition education interventions should actively engage both women and men to encourage shared household decision‐making regarding food purchasing and preparation. Couple‐based nutrition counseling during antenatal care, community dialogue sessions involving husbands, and behavior change communication delivered through health extension workers may help improve shared decision‐making regarding food purchasing, preparation, and appropriate iodized salt utilization. Community‐based health education delivered through health extension workers and antenatal care services may provide an opportunity to promote equitable household participation in nutrition‐related decisions.

Importantly, good knowledge of iodized salt was strongly associated with better utilization practices, which is consistent with the theoretical expectation that awareness influences behavior. Although the posterior estimate demonstrated a strong association, the relatively wide credible interval indicates some imprecision in the magnitude of effect. Therefore, emphasis should be placed on the direction rather than the exact size of the association. This finding reinforces the importance of health education interventions aimed at improving knowledge as a pathway to enhancing proper utilization practices. However, the persistence of a gap between knowledge and practice suggests that structural and contextual barriers must also be addressed. Notably, although knowledge significantly predicted utilization practices, it was not significantly associated with household availability of adequately iodized salt after adjustment for other variables. This finding suggests that adequate household iodine content depends not only on knowledge but also on external factors such as market availability, affordability, product quality, and regulatory enforcement.

Households with larger family sizes were inversely associated with the availability of adequately iodized salt, meaning that larger households had lower odds of having adequately iodized salt. This may be due to higher consumption rates, which can lead to more frequent purchases of cheaper or inadequately iodized salt, as well as difficulties in maintaining proper storage practices over time. This finding highlights the importance of considering household dynamics in interventions aimed at improving iodized salt utilization.

### 4.1. Strengths and Limitations of the Study

#### 4.1.1. Strengths

This study has several important strengths that enhance the validity and relevance of its findings. First, the study employed a community‐based design, which enhances the representativeness of the findings for the source population of pregnant women in Awash Sebat Administrative City. Unlike facility‐based studies, this approach captured women regardless of health service utilization, thereby reducing selection bias and providing a more accurate reflection of household iodized salt knowledge, utilization practices, and availability. Second, the use of a probability sampling technique (simple random sampling with proportional allocation) ensured that each eligible pregnant woman had an equal probability of selection, thereby minimizing sampling bias. Third, the application of Bayesian logistic regression allowed the incorporation of weakly informative priors and estimation of posterior credible intervals, providing robust parameter estimates while accounting for uncertainty in the associations. Moreover, sensitivity analyses were conducted and demonstrated robust findings. Finally, household salt iodine content was assessed using a standardized RTK rather than relying solely on participants′ self‐reports, improving the objectivity of the measurement.

#### 4.1.2. Limitations

Despite these strengths, the study has some limitations. First, because of its cross‐sectional design, temporal relationships cannot be established, and the observed associations should not be interpreted as causal. Second, although the study was community‐based, the vast majority of participants were urban residents, with rural and pastoral women being underrepresented. Consequently, the findings should not be generalized to predominantly rural and pastoral populations in the Afar Region, where access to iodized salt and utilization practices may differ. Third, the sampling frame was based on health extension worker registration lists, which may have excluded unregistered pregnant women and introduced selection bias. Fourth, household salt iodine content was assessed using a RTK, which has limited specificity and may have resulted in some misclassification of adequately iodized salt, potentially affecting the estimated prevalence. Finally, some regression estimates had relatively wide credible intervals, likely reflecting small sample sizes in certain subgroups, and these associations should therefore be interpreted with appropriate caution.

## 5. Future Research Recommendations

Future studies should include larger and more representative rural and pastoral populations to improve generalizability across the Afar Region. Longitudinal studies are needed to establish causal relationships between knowledge, utilization practices, and household iodized salt adequacy. Future research should also evaluate the performance of RTKs against laboratory‐based iodine measurements and examine the use of locally produced coarse salt, the quality and consistency of information delivered through different communication channels, market availability, affordability, and behavioral factors influencing household iodized salt utilization. Qualitative and mixed‐methods studies would further help explain the observed knowledge–practice gap and the contextual factors underlying household decision‐making.

## 6. Programmatic Implications

Strengthening regulatory monitoring and enforcement of USI throughout salt production, transportation, wholesale distribution, retail markets, and household supply chains remains essential, particularly in pastoral regions where packaged adequately iodized salt may be less accessible than locally produced coarse salt. Health education interventions should move beyond improving knowledge alone by addressing behavioral barriers that prevent appropriate utilization practices. Community‐based nutrition education should actively involve husbands and other household decision‐makers to encourage shared decision‐making regarding food purchasing and preparation. Targeted interventions for larger households and rural pastoral communities may also improve equitable access to adequately iodized salt. Furthermore, strengthening collaboration between health extension workers, local markets, and regulatory agencies could improve both the availability and appropriate utilization of adequately iodized salt. Nutrition education should also ensure that communication messages consistently emphasize both the importance of consuming adequately iodized salt and the specific health consequences of IDDs, while promoting the purchase of adequately iodized packaged salt instead of locally available non‐iodized coarse salt where applicable.

## 7. Conclusion

Although knowledge of iodized salt among pregnant women was relatively high, utilization practices and the availability of adequately iodized salt were lower, indicating persistent gaps. The proportion of adequately iodized salt remains below the World Health Organization target for USI, highlighting an important public health concern for maternal nutrition and the prevention of IDDs during pregnancy. Higher maternal education, urban residence, and better household wealth were consistently associated with improved knowledge, utilization practices, and adequate iodized salt availability. Good knowledge was also significantly associated with better utilization practices, although it was not significantly associated with household availability of adequately iodized salt, suggesting that improved knowledge alone may not overcome barriers related to access, affordability, or the quality of salt available in the market. In contrast, husband‐only decision‐making was associated with poorer utilization practices, while larger household size was associated with lower odds of having adequately iodized salt.

These findings suggest that improving iodine nutrition among pregnant women will require coordinated interventions that extend beyond health education alone. Policy efforts should strengthen enforcement and routine monitoring of salt iodization standards across the supply chain, improve the availability and affordability of adequately iodized packaged salt, particularly in underserved communities, and reinforce antenatal counseling on appropriate iodized salt storage and utilization. Community‐based interventions that encourage joint household decision‐making and actively engage husbands and other family members may also help translate knowledge into appropriate utilization practices.

Because this study was conducted predominantly among urban residents of Awash Sebat Administrative City, the findings should be interpreted primarily within this context and should not be generalized to the wider rural and pastoral populations of the Afar Region, where access to iodized salt and utilization practices may differ substantially. Future studies should include better representation of rural pastoral communities and use longitudinal or implementation‐focused designs to evaluate strategies for improving access to adequately iodized salt and reducing iodine deficiency among pregnant women.

**Nomenclature**
WHO: World Health Organization
DHS: Demographic and Health Survey
Fig.: Figure
VIF: Variance inflation factor
MCMC: Markov chain Monte Carlo
MCSE: Monte Carlo standard error
OR: Odds ratio
Std. Dev.: Standard deviation
CrI: Credible interval

## Author Contributions

Temesgen Gebeyehu Wondmeneh and Asiya Hussein Ibrahim conceived and conducted the study, performed the data analysis, and drafted the manuscript. They also interpreted the findings and critically revised the manuscript.

## Funding

The authors received no financial support for the research, authorship, or publication of this article.

## Disclosure

All authors read and approved the final version of the manuscript.

## Ethics Statement

The proposal was ethically reviewed and approved by the Institutional Ethical Review Committee (IERC) of Samara University, College of Medicine and Health Sciences (Ref. No: SU/IRB/38/35/814/2016). A letter of cooperation was obtained from Samara University and submitted to the Awash Health Administration Office, and permission was secured through the appropriate administrative channels. Kebele administrators, in collaboration with health extension workers, were informed about the study and assisted in identifying eligible participants. Before data collection, participants were informed of the study’s purpose, importance, and duration. Written informed consent was obtained prior to the interview. Confidentiality and anonymity were ensured using anonymous questionnaires without personal identifiers. Participation was voluntary, and participants could refuse or withdraw at any time without consequence.

## Consent

The authors have nothing to report.

## Conflicts of Interest

The authors declare no conflicts of interest.

## Supporting Information

Additional supporting information can be found online in the Supporting Information section.

## Supporting information


**Supporting Information 1** S Figures 1–6 Markov chain Monte Carlo (MCMC) diagnostic plots (trace, histogram, autocorrelation, and posterior density plots) for knowledge of iodized salt.

**Supporting Information 2** S Figures 7–13 Markov chain Monte Carlo (MCMC) diagnostic plots (trace, histogram, autocorrelation, and posterior density plots) for iodized salt utilization practices.

**Supporting Information 3** S Figures 14–21 Markov chain Monte Carlo (MCMC) diagnostic plots (trace, histogram, autocorrelation, and posterior density plots) for the adequacy of iodized salt.

## Data Availability

All relevant data are included in the manuscript. If additional data are requested, the corresponding author is willing to provide them or clarify any ambiguities.
