# Supplementary material for: Knowledge, Utilization Practices, and Adequacy of Iodized Salt Among Pregnant Women in Awash Sebat Administrative City, Afar Region, Ethiopia: A Bayesian Logistic Regression Analysis
Source: J Nutr Metab. 2026 Sep 2;2026:1557188. doi: 10.1155/jnme/1557188 (PMC13535854; doi:10.1155/jnme/1557188)
Supplement: Supplementary file 3 [file JNME-2026-1557188-s003.zip › S Fig.9.docx]

S Fig.9 Markov Chain Monte Carlo diagnostic plots (trace, histogram, autocorrelation, and posterior density plots) for utilization practice of iodine salt and mothers’ ages
